# The association between telomerase activity and expression of its RNA component (hTR) in breast cancer patients: the importance of DNase treatment

**DOI:** 10.1186/1477-3163-5-17

**Published:** 2006-06-02

**Authors:** Saied Hosseini-Asl, Mohammad H Modarressi, Morteza Atri, Mohamed Salhab, Kefah Mokbel, Parvin Mehdipour

**Affiliations:** 1 Department of Medical Genetics, Faculty of Medicine, Tehran University of Medical Sciences, Tehran, IR, Iran; 2 Cancer Institute, Tehran University of Medical Sciences/Day Hospital, Tehran, IR, Iran; 3 St. George's Hospital, London, SW17 0QT, UK

## Abstract

Telomerase is a ribonucleoprotein enzyme that compensates for the telomere length shortening which occurs during the cell cycle. Telomerase activity has been detected in most tumours but not in somatic cells. However, hTR; the RNA component of telomerase; has been reported to be universally expressed in both cancerous and non-cancerous tissues. Tumour samples from 50 patients with primary invasive breast cancer were collected. The TRAP assay was used to detect telomerase activity. RT-PCR on cDNA and DNased cDNA samples and control groups was used to detect the expression of hTR, GAPDH and PGM1 genes. Seventy-two percent of samples showed telomerase activity. DNA contamination was detected in 36 (72%) of RNA samples. Without performing DNase treatment, 49 (98%) of all samples showed hTR expression, but with the application of this strategy, hTR expression decreased from 98% to 64%. A significant association (p < 0.001) between hTR expression and telomerase activity was observed. Among the 32 hTR positive samples, 30 had telomerase activity and among the 18 hTR negative samples, telomerase activity was observed in 6 cases. Thus the application of this strategy could provide an applicable tool to use instead of the TRAP assay thus facilitating telomerase research in cancer genetic investigations.

## Introduction

Telomerase is a ribonucleoprotein that compensates the shortening of the ends of eukaryotic chromosomes occurring during the cell cycle. This RNA-dependent DNA polymerase provides the basis for an unlimited proliferation. Its activity is absent in most normal human somatic cells, including breast tissue, but is present in over 90% of cancerous cells and in vitro-immortalized cells [1-14]. Telomerase consists of two fundamental components, an RNA component (in humans, hTR or hTERc) and a reverse transcriptase component (hTERT) [15].

The RNA component of telomerase acts as the template for telomeric repeat synthesis. In man, hTR is transcribed by RNA polymerase II and its mature transcript consists of 451 nucleotides [16]. The hTR gene was cloned and localized to chromosome 3q26.3. in 1998 [17,18]. The template for hTERT activity lies in nucleotides 46 to 53. Although there is a variation of hTR RNA sequences among telomerase RNAs, there is a remarkably conserved secondary structure from ciliates to vertebrates. This indicates an essential role for the structure in enzyme function [19]. hTR is a single-copy gene that lacks poly A and does not contain any introns, so RT-PCR for hTR gene is considered to be prone to errors. DNA contamination of RNA extractions could be amplified by PCR and therefore could give rise to a false positive result for hTR transcription affecting the correlation between hTR expression and telomerase activity [20,21]

## Materials and methods

### Patients

Institutional guidelines including ethical approval and informed consent were followed. We investigated 50 tumour samples from patients with primary invasive breast cancer treated surgically during 2004–2005 at Tehran University of Medical Sciences. Breast tissues were collected and preserved by rapid freezing in liquid nitrogen immediately after surgical excision and then were stored at -70°C.

### The TRAP assay

Telomerase activity was analyzed using the PCR-based telomeric-repeat amplification protocol (TRAP assay) as previously described [22].

The frozen samples (50 mg) were homogenized in 100 μl chaps lysis buffer (10 mM Tris-HCl (pH 8.3), 1 mM MgCl_2_, 1 mM EGTA, 5 mM β-mercaptoethanol, 0.5% CHAPS, 10% Glycerol and 0.1 mM PMSF (β-mercaptoethanol and PMSF were added before use).

After incubation for 30 minutes on ice, the lysate was centrifuged for 20 min at 12,000 g at 4°C, and then, the supernatant was immediately stored at -70°C. Aliquots of 0.5 to 5 μg of protein were incubated with 30 μl of a reaction mixture containing 20 mM Tris-HCl (pH 8.3), 1.5 mM MgCl_2_, 63 mM KCl, 0.05% Tween 20, 1 mM EGTA (TitriplexVI, Merck, USA), 125 μM dNTPs and 50 ng of TS primer (5'- AATCCGTCGAGCAGAGTT-3') in a thermocycler for 35 min at 30°C for generation of telomeric repeats (presence or absence of T4gene32protein did not affect the results.). To avoid probable RNase activity, mineral oil was added in the last minuets, by denaturation at 94°C for 5 min (for inactivating telomerase activity) and preserving at 70°C, 2.5 U Taq DNA polymerase (Roche, Germany), 50 ng of ACX primer (5'-GCGCGG(CTTACC)_3_CTAACC-3'), 50 ng of NT internal control primer (5'-ATCGCTTCTCGGCCTTTT-3') and 1.3 × 10^-9^ng of TSNT internal control (5'-AATCCGTCGAGCGCAGAGGTTAAAAGGCCGAGAAGCGAT-3') were added. Then, the mixture was subjected to 35 PCR cycles at 94°C for 30 s, 56°C for 45 s, and 72°C for 45 s.

The PCR products were mixed with a loading dye and electrophoresed on 10% non-denaturing polyacrylamide gel, and finally, stained by silver nitrate which has more sensitivity than SYBR Green and Ethidium bromide.

**Figure 1:**
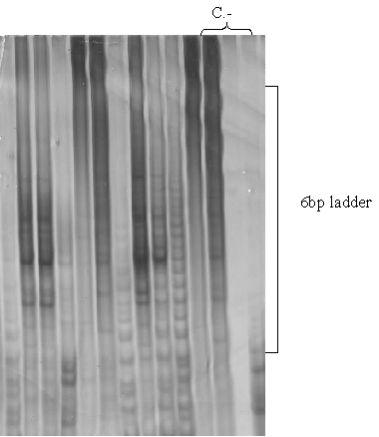
**Telomerase activity detected by TRAP assay method**. C.-: A negative control group including a telomerase negative sample, a denatured protein sample and a mixture without protein extract.

An internal control was used to detect false-negative samples containing PCR inhibitors (such as CHAPS). In order to avoid false-positive results (characterized by variation in band intensity), negative controls were used. It was produced by heating the protein extract for 5 min at 90–95°C (to remove the enzyme activity) or using any telomerase negative sample (Fig. 1).

### RT-PCR analysis

Total RNA was isolated from samples using Tripure Isolation Reagent (Roche, Germany). One microgram of total RNA, with random hexamer and M-Mulv reverse transcriptase enzyme (Fermentas co, Canada.) were used to create cDNA for each sample, according to the manufacturer's protocol. In order to avoid he probable DNA contamination for RNA samples, the following stages were performed. We prepared a solution containing the same materials used for cDNA synthesis excluding reverse transcriptase enzyme (negative control 1). This product contains DNA only, with a new concentration similar to the cDNA products, however, in the PCR products of these samples, the presence or absence of any DNA contamination could be observed and detected.

In order to perform DNase treatment, 1 μg of total RNA was digested by DNaseI (Fermentas Co, Canada) according to the manufacturer's protocol. Half of DNase treated RNA sample was used to create cDNA. The remaining half of the sample contained all of the materials excluding the reverse transcriptase enzyme (negative control 2), in order to validate the accuracy of DNase treatment process.

The cDNA, DNase treated cDNA and two control group samples were amplified in a 25 μl reaction mixture containing 0.2 μM of each primers and 1U Taq DNA polymerase (Fermentas co., Canada). GAPDH was amplified using GapF (5'-GGGAAGGT GAAGGTCGGAGTC-3') and GapR (5'-AGCAGAGGGGGCAGA GATGAT-3') oligonucleotides with an initial heating at 95°C for 3 min, followed by 30 cycles of 94°C for 45 s, 63°C for 45 s, and 72°C for 45 s. Due to the existence of GAPDH pseudogene and its amplification in DNA samples as well as cDNA, and to avoid its false positive results (will be explained in discussion), the PGM1 (Phosphoglucomutase1) a housekeeping gene was used as a control which could detect the presence of cDNA in the samples. PGM1 was amplified using PGM1-F (5'-TCCGACTGAGCGGCACTGGGAGTGC-3')and PGM1-R (GCCCGCAGGTCC TCTTTCCCTCACA-3') oligonucleotides with 30 cycles of 94°C for 30 s, 63°C for 30 s, and 72°C for 30 s. hTR was amplified using TR-F (5'-CGCCGTGCTTTTGCT CC-3') and TR-R (5'-ACTCGCTCCGTTCCTCTTCC-3') oligonucleotides for 5 cycles of 94°C for 1 min, 63°C for 1 min, and 72°C for 1 min, followed by 30 cycles of 94°C for 45 s, 63°C for 50 s, and 72°C for 45 s. Amplified products were subjected to electrophoresis in 2% agarose gels and were visualized with ethidium bromide.

### Statistical analysis

The statistical analysis of the data was carried out by using the SPSS software package (SPSS Inc; Chicago, IL, USA; Version 11.5, 2003). The Pearson, Chi-square and Fisher Exact tests were used. The significance levels were considered for results with P value less than 0.05.

## Results

Telomerase activity was detected in 36 out of 50 (72%) samples (Table 1). The hTR gene expression was observed in 32 (64%) samples of which 30 (93.7%) samples had telomerase activity. In 6 (33%) samples without hTR expression, telomerase activity was detected (Table 2). There was a significant association between telomerase activity and hTR expression (Chi square = 20.85, p <0.001).

**Table 1:** The characteristic results of the TRAP assay and RT-PCR on PGM1, GAPDH and hTR genes.

		PGM1		GAPDH				hTR			
				
No.	Telomerase activity	cDNA	DNased cDNA	cDNA	RNA withoutRT*	DNased cDNA	DN-RNA withoutRT**	cDNA	RNA withoutRT*	DNased cDNA	DN-RNA withoutRT**
			
1	+	+	+	+	+	+	-	+	+	+	-
2	+	+	+	+	+	+	-	+	+	-	-
3	+	+	+	+	+	+	-	+	+	+	-
4	+	+	+	+	-	+	-	+	-	+	-
5	+	+	+	+	+	+	-	+	+	+	-
6	-	+	+	+	+	+	-	+	+	-	-
7	-	+	+	+	+	+	-	+	+	+	-
8	+	+	+	+	+	+	-	+	+	+	-
9	-	+	+	+	+	+	-	+	+	-	-
10	+	+	+	+	-	+	-	+	-	+	-
11	+	+	+	+	+	+	-	+	+	-	-
12	+	+	+	+	+	+	-	+	+	-	-
13	+	+	+	+	-	+	-	+	-	+	-
14	+	+	+	+	-	+	-	-	-	-	-
15	+	+	+	+	+	+	-	+	+	+	-
16	+	+	+	+	+	+	-	+	+	+	-
17	+	+	+	+	+	+	-	+	+	+	-
18	-	+	+	+	+	+	-	+	+	-	-
19	-	+	+	+	+	+	-	+	+	-	-
20	-	+	+	+	+	+	-	+	+	-	-
21	+	+	+	+	+	+	-	+	+	+	-
22	+	+	+	+	-	+	-	+	-	+	-
23	-	+	+	+	+	+	-	+	+	-	-
24	+	+	+	+	-	+	-	+	-	+	-
25	+	+	+	+	+	+	-	+	+	+	-
26	+	+	+	+	-	+	-	+	-	+	-
27	+	+	+	+	+	+	-	+	+	-	-
28	-	+	+	+	-	+	-	+	-	+	-
29	+	+	+	+	-	+	-	+	-	+	-
30	-	+	+	+	+	+	-	+	+	-	-
31	+	+	+	+	-	+	-	+	-	+	-
32	-	+	+	+	+	+	-	+	+	-	-
33	+	+	+	+	-	+	-	+	-	+	-
34	-	+	+	+	+	+	-	+	+	-	-
35	-	+	+	+	+	+	-	+	+	-	-
36	-	+	+	+	+	+	-	+	+	-	-
37	+	+	+	+	+	+	-	+	+	+	-
38	+	+	+	+	-	+	-	+	-	+	-
39	+	+	+	+	+	+	-	+	+	+	-
40	+	+	+	+	+	+	-	+	+	+	-
41	+	+	+	+	+	+	-	+	+	+	-
42	+	+	+	+	-	+	-	+	-	+	-
43	+	+	+	+	+	+	-	+	+	-	-
44	+	+	+	+	+	+	-	+	+	+	-
45	+	+	+	+	+	+	-	+	+	+	-
46	+	+	+	+	+	+	-	+	+	+	-
47	+	+	+	+	+	+	-	+	+	+	-
48	+	+	+	+	-	+	-	+	-	+	-
49	+	+	+	+	+	+	-	+	+	+	-
50	-	+	+	+	+	+	-	+	+	-	-

*: Mixture of RNA and materials required for cDNA synthesis without Reverse Transcriptase enzyme, determining the probable DNA contamination.

**: Mixture of DNased RNA and materials required for cDNA synthesis without Reverse Transcriptase enzyme, determining the accuracy of DNase treatment.

**Table 2:** The frequency of telomerase activity between either sample with hTR expression or without expression.

	**HTR expression**		
	
**Telomerase activity**	Positive	Negative	Total
Positive	60% (30)*	12% (6)	72% (36)
Negative	4% (2)	24% (12)	28% (14)
Total	64% (32)	36% (18)	50

*The outcomes presented as percentage (number). Chi-square = 20.85, P < 0.001

By using GAPDH primers in the control products (negative control groups 1 and 2), it was observed that 36 (72%) RNA extracts had DNA contamination. In total, 36 samples (with DNA contamination) demonstrated hTR expression in cDNA samples, whereas after DNase treatment, 17 samples did not show hTR expression.

The hTR expression in cDNAs was observed in 49 out of 50 (98%) samples, of which, 35 had telomerase activity. In one case without any DNA contamination and with telomerase activity, hTR expression in cDNA and DNased cDNA samples was not observed.

In cDNA samples with negative results for GAPDH-PCR, the hTR – PCR was found to be negative, which confirms the lack of any DNA contamination (Table 1).

## Discussion

**Figure 2:**
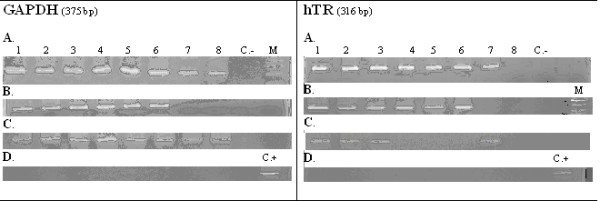
**Electrophoregram for GAPDH and hTR PCR products**. **A**: cDNA samples. **B**: Mixture of RNA and materials required for cDNA synthesis excluding Reverse Transcriptase enzyme, determining the probable DNA contamination. **C**: DNase treated cDNA. **D**: Mixture of DNased RNA and required for cDNA synthesis excluding Reverse Transcriptase enzyme, determining the accuracy of DNase treatment. All of samples had GAPDH expression but lanes 7 and 8 did not have expression of this gene (in RNA-RT samples) and so did not have DNA contamination. Lanes 1–3 had positive expression of hTR. Lanes 4–6 did not have expression on DNased treated samples and so were negative but if we had not used DNase treatment, the expression was determined and this was caused by DNA contamination. Lane 7 did not have DNA contamination and the band seen in PCR for cDNA sample does not belong to DNA contamination. Lane 8 did not have any DNA contamination and hTR expression, too. C.-: Negative control including any cDNA or DNA template. C. +: Positive control (a template with positive expression of gene) to reach the accuracy of PCR. M: DNA size marker.

GAPDH has a psuedogene which could be amplified with GAPDH cDNA primers, as well as the current GAPDH gene cDNA. Usually, RNA is contaminated with DNA and due to the performance of PCR on a negative control group (this product has only probable DNA contamination), it will create a positive result that arises from the presence of a pseudogene in the sample(Fig. 2). The present subject was not considered in most studies in which the GAPDH housekeeping gene was used as a positive control to assess the accuracy of cDNA synthesis. In fact, the positive result of GAPDH-PCR may be due to the amplification of the pseudogene and not to the cDNA template. If GAPDH primers are used for DNA samples, then positive results will be occur. In this regard, this characteristic of GAPDH was used to ensure the accuracy of DNase treatment procedures.

When the result of GAPDH-PCR was positive in DNased treated cDNA samples, and negative in negative control group 2, then it can be assumed that the existing templates in DNased treated samples are cDNA and are not the result of any DNA contamination.

The inactivation of DNase enzyme seems to be another problem in DNase treatment procedures. If a high temperature is used for denaturation of the enzyme, this would affect the RNA molecules, and if DNase enzyme denaturation is not completely performed, it would have an effect on PCR performance of the samples. In the present investigation, all of the treated samples with DNase enzyme were checked with GAPDH as well as with PGM1 housekeeping genes for detecting the presence of cDNA and were, finally, subjected to PCR for the hTR gene.

Most investigators [2-4,11-13,23-27] have not paid any attention to the lack of intron of hTR gene. Therefore, the reported results were representative of a high expression of this gene and hence no association could be observed between hTR expression and telomerase activity (Table 3).

**Table 3:** The results of some previous studies on hTR expression using RT-PCR method without DNase treatment.

**HTR expression**	**Type of cancer studied**	**Ref**.
58/62 (93.6%)	Skin neoplasm	23
26/26 (100%)	Stomach cancer	2
55/55 (100%)	32 normal human Endometrial and 23 endometrial cancers	3
18/18 (100%)	Prostate	4
34/34 (100%)	Colonic cancer	11
11/11 (100%)	Parathyroid lesions	12
20/20 (100%)	Renal cell carcinoma	13
20/20 (100%)	Ovarian adenocarcinoma	24
39/43 (90.7%)	Nasopharyngeal carcinoma	25
42/45 (93.3%)	Skin tumors	26
39/39 (100%)*	Colorectal carcinoma	27*

*: DNase treatment was performed, but, the accuracy of DNase digestion and probable effect of remaining DNase activity on the genes PCR had not assayed.

Furthermore, the effect of the probable remaining DNase activity post DNase enzyme inactivation was not noticed in previous studies using DNase treatment [27].

In this study, the accuracy of the procedures, including DNase treatment and cDNA synthesis and creating negative control groups were checked with GAPDH and PGM1 housekeeping genes. In the case of having a positive result for negative control groups, or a negative result for DNase treated cDNAs, then the repeat of the DNase treatment and use of negative control groups would be required.

A false estimation of our result, through a procedure without DNase treatment, would be expected, i.e., instead of observing 49 (98%) with hTR expression, the outcome could be decreased to 64%.

## References

[B1] Kim NW, Piatyszek MA, Prowse KR, Harley CB, West MD, Ho PL, Coviello GM, Wright WE, Weinrich SL, Shay JW (1994). Specific association of human telomerase activity with immortal cells and cancer. Science.

[B2] Kuniyasu H, Domen T, Hamamoto T, Yokozaki H, Yasui W, Tahara H, Tahara F (1997). Expression of human telomerase RNA in an early event of stomach carcinogenesis. Jpn J Cancer.

[B3] Kyo S, Kanaya M, Takakura M, Tanaka M, Inoue M (1999). Human telomerase reverse transcriptase as a critical determinant of telomerase activity in normal and malignant endometrial tissues. Int J Cancer.

[B4] Liu BC-S, Larose I, Weinstein LJ, Ahn M, Weinstein MH, Richie JP (2001). Expression of telomerse subunits in normal and neoplastic prostate epithelial cells isolated by laser capture microdisection. Am Cancer Soc.

[B5] Loveday RL, Greenman J, Drew PJ, Monson JRT, Kerin MJ (1999). Genetic changes associated with telomerase activity in breast cancer. Int J Cancer.

[B6] Mauro LJ, Foater DN (2002). Regulators of telomerase activity. Cell Mol Biol.

[B7] Mokbel K, Parris CN, Radbourne R, Ghilchik M, Newbold F (1999). Telomerase activity and breast cancer. Eur J Surg Oncol.

[B8] Nakamura A, Suda T, Honma T, Takahashi T, Igarashi M, Waguri N, Kawai H, Mita Y, Aoyagi Y (2004). Increased hTR expression during transition from adenoma to carcinoma is not associated with promoter methylation. Dig Dis Sci.

[B9] Newbold RF (2002). The significance of Telomerase activation and cellular immortalization in human cancer. Mutagenesis.

[B10] Novakovic S, Hocevar M, Zgajnar J, Besic N, Stegel V (2004). Detection of telomerse RNA in the plasma of patients with breast cancer, malignant melanoma or thyroid cancer. Oncol Rep.

[B11] Nowak J, Januszkiewicz D, Lewandowski K, Nowicka K, Pernak M, Rembowska J, Nowak T, Wysocki J (2003). Activity and expression of human telomerase in normal and malignant cells in gastric and colon cancer patients. Eur J Cast Hepathol.

[B12] Onada N, Ogisawa K, Ishikawa T, Takenaka C, Tahara H, Inaba M, Takashima T, Hirakawa K (2004). Telomerase activation and expression of its catalytic subunits in benign and malignant tumors of the parathyroid. Surg Today.

[B13] Rohde V, Sattler HP, Bund T, Bonkhoff H, Fixemer T, Bachmann C, Lensch R, Unteregger G, Stoeckle M, Wullich B (2000). Expression of the human telomerase reverse transcriptase is not related to telomerase activity in normal malignant renal tissue. Clin Cancer Res.

[B14] Shay JW, Bacchetti S (1997). A survey of telomerase activity in human cancer. Eur J Cancer.

[B15] Bettendorf O, Heine B, Kneif S, Eltze E, Semjonow A, Herbst H, Stein H, Bocker W, Poremba C (2003). Expression-patterns of the RNA component (hTR) and the catalytic subunit (hTERT) of human telomerase in non-neoplastic prostate tissue, prostatic intraepithelial neoplasia, and prostate cancer. Prostate.

[B16] Feng J, Eunk WD, Wang SS, Weinrich SI, Avilion AA, Chiu CP, Adams RK, Chang E (1995). The RNA component of human telomerase. Science.

[B17] Soder AI, Hoare SF, Murie S, Balmain A, Parkonson EK, Keith WN (1997). Mapping of the gene for the mouse telomerase RNA component, Terc, chromosome painting. Genomics.

[B18] Zhao JQ, Hoare SF, McFarlane R, Muir S, Parkinson EK, Black DM, Keith WN (1998). Cloning and characterization of human and mouse telomerase RNA gene promoter sequences. Oncogene.

[B19] Chen JL, Blasco MA, Greider CW (2000). Secondary structure of vertebrate telomerase RNA. Cell.

[B20] Januszkiewicz D, Wyoski J, Lewandowski K, Pernak M, Nowicka K (2003). Lack of correlation between telomere length and telomerase activity and expression in leukemic cells. Int J Mol Med.

[B21] Kameshima H, Yagihashi A, Yajima T, Kobayashi D, Hirata K, Watanabe N (2001). Expression of telomerase-associated genes: reflection of telomerase activity in gastric cancer?. World J Surg.

[B22] Bachand F, Triki I, Autexier C (2001). Human telomerase RNA-protein interactions. Nuc Acids Res.

[B23] Hu S, Chan HL, Chen MC, Pang JH (2002). Telomerase expression in benign and malignant skin neoplasms: comparison of three major subunits. J Formos Med Assoc.

[B24] Ulaner GA, Hu JF, Vu TH, Oruganti H, Giudice LC, Hoffman AR (2000). Regulation of telomerase by alternate splicing of human telomerase reverse transcriptase (hTERT) in normal and neoplastic ovary, endometrium and myometrium. Int J Cancer.

[B25] Wang X, Xiao J, Zhao S, Tian Y, Wang G (2001). Expression of telomerase subunits and its relationship with telomerase activity in nasophryngeal carcinoma. Zhongua Bing Li Xue Za Zhi.

[B26] Wu A, Ichihashi M, Ueda M (1999). Correlation of the human telomerase subunits with telomerase activity in normal skin and skin tumors. Am Cancer Soc.

[B27] Yan P, Saraga EP, Bouzourene H, Bosman FT, Benhattar J (2001). Expression of telomerase genes correlates with telomerase activity in human colorectal carcinogenesis. J Pathol.

